# Brain abscess in a patient with psoriatic arthritis treated with adalimumab: A case report

**DOI:** 10.1097/MD.0000000000018954

**Published:** 2020-03-06

**Authors:** Yu-Pei Lo, Snehal Desale, Po-Yuan Wu

**Affiliations:** a Department of Dermatology, China Medical University Hospital; b Department of Dermatology, China Medical University, Taiwan, ROC.

**Keywords:** adalimumab, odontogenic brain abscess, psoriatic arthritis

## Abstract

**Rationale:**

In patients receiving biological therapies, serious infections are a major concern. Infections associated with anti-tumor necrosis factor antibody therapy include tuberculosis, viral, fungal, and bacterial infections. Likewise, severe infections of the upper and lower respiratory tract, lung, skin and soft tissue, urinary tract, gastrointestinal tract, joint, and bone have also been reported previously. However, infections involving the central nervous system are rare, especially an intracranial infection caused by odontogenic infection. To date, only few cases have been reported of this infection. This is the first case of a patient with psoriatic arthritis receiving adalimumab and developing brain abscess of odontogenic origin.

**Patient concerns:**

A 39-year-old male with psoriatic arthritis receiving adalimumab treatment came to the emergency department with initial presentation of sudden onset convulsions. He had been receiving adalimumab treatment for 1 month. Two days after the third injection, the patient had an episode of sudden-onset general convulsion for nearly 5 min with the upgazing and general tonic presentation. Magnetic resonance imaging (MRI) showed left frontal lobe brain abscess. Pus culture from the brain abscess detected *Streptococcus sanguinis* (*S. sanguinis*), *Fusobacterium nucleatum* (*F. nucleatum*), and *Parvimonas micra* (*P. micra*).

**Diagnosis:**

Brain abscess with odontogenic infection.

**Interventions:**

The patient received left frontal craniotomy, abscess drainage and systemic empiric antibiotics treatment with vancomycin, cefepime, and metronidazole. Due to drug rash with eosinophilia and systemic symptoms during the treatment, vancomycin and metronidazole were discontinued, and systemic antibiotics were switched to teicoplanin and ceftriaxone.

**Outcomes:**

A brain MRI follow-up performed after 1 month of initial treatment revealed the reduced size of the abscess lesion and minimal oedema. The patient was discharged with stable condition.

**Lessons:**

To the best of our knowledge, this is the first case of a patient with psoriatic arthritis receiving adalimumab and developing brain abscess of odontogenic origin. Such a rare diagnosis must be kept in mind when patients treated with adalimumab present with sudden-onset convulsions. Careful dental examination should be performed before administration of adalimumab.

## 1 Introduction

Adalimumab is an anti-tumor necrosis factor (TNF) alpha monoclonal antibody used to treat psoriatic arthritis. This biological agent poses an increased risk for opportunistic infection of bacterial, viral, or fungal origins. Tuberculosis is commonly found due to immune suppression.^[1]^ Cerebral infections are very rarely reported in patients treated with adalimumab. Brain abscess is diagnosed in our case after receiving three doses of adalimumab with the initial presentation of general convulsions. MRI image revealed left frontal lobe brain abscess. Pus culture detected *Streptococcus sanguinis* (*S. sanguinis*), *Fusobacterium nucleatum* (*F. nucleatum*), and *Parvimonas micra* (*P. micra*), confirming odontogenic nature of the brain abscess. This is the first case of psoriatic arthritis receiving adalimumab complicated by odontogenic brain abscess.

## 2 Case report

**Figure 1:**
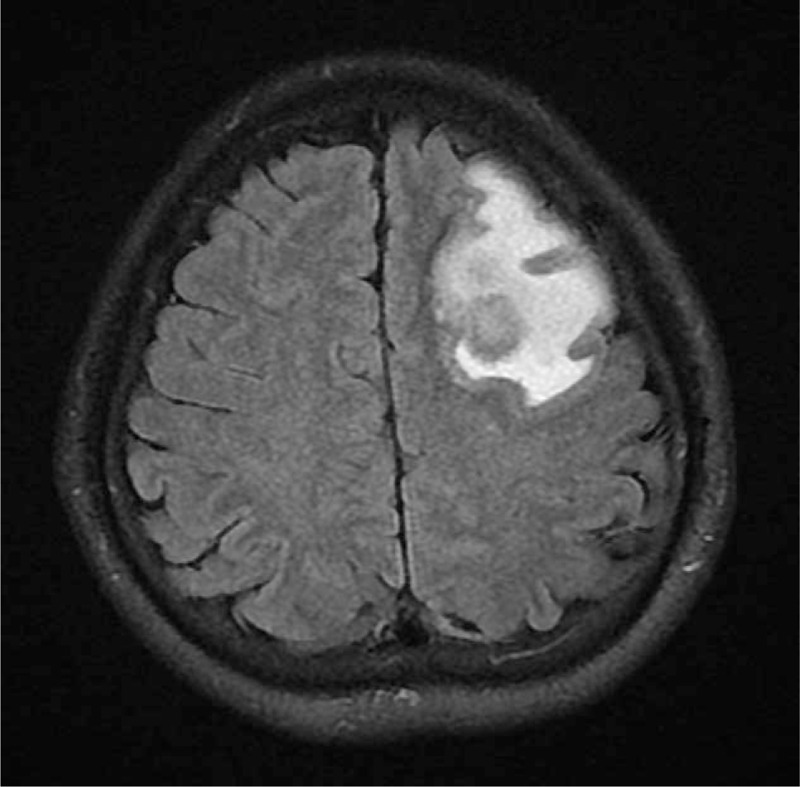
Brain magnetic resonance imaging revealed left frontal lobe abscess.

A 39-year-old male with a past history of psoriatic arthritis was referred to our emergency department because of sudden-onset general convulsion. Two years ago, the patient was diagnosed as psoriatic arthritis. The patient had been receiving treatment with topical Calcipotriol, oral Methotrexate, and Cyclosporine. The patient had general malaise after taking Methotrexate. He was then switched to adalimumab treatment for 1 month. Two days after the third injection, the patient had an episode of sudden-onset general convulsion for nearly 5 minutes with the upgazing and general tonic presentation. At our emergency department, physical examination findings were as follows: body temperature, 36.7°C; blood pressure, 139/90 mm Hg; heart rate, 86 beats/min; and respiratory rate, 19 breaths/min. We noted erythematous well-defined scaly patches and plaques at the trunk and limbs. The neurological examination revealed neither central nervous system (CNS) deficits nor focal deficits. Laboratory tests revealed the following: the complete blood count was notable for leucocytosis, 12200/mm^3^; neutrophils segment, 57.1%; hemoglobin, 16.5 g/dL; haematocrit, 47.6%; and platelet count, 369,000/μL. A serum blood test revealed hypokalaemia (3.3 mmol/L) and the glucose level at 128 mg/dL. Conversely, an Human Immunodeficiency Virus (HIV) antibody screening test was negative, as were urine, blood, and stool culture tests. Computed tomography (CT) revealed a left-side frontal lobe ring-enhancement lesion with perifocal edema. Furthermore, brain MRI detected left frontal lobe abscess (Fig. 1). On admission, the patient underwent left frontal craniotomy and abscess drainage. Initially, the patient was prescribed systemic antibiotics with vancomycin, cefepime and metronidazole. Pus culture from the brain abscess detected *S. sanguinis*, *F. nucleatum*, and *P. micra*. Unfortunately, after 1 week, the patient developed generalised erythematous patches, macules and scaly plaques on the face, scalp, trunk, four limbs, palms, and soles. Skin lesions were accompanied by facial swelling, high fever (up to 39.7°C), lymphocyte below normal limits (lymphocytes: 9.7%) and increased liver enzyme level (aspartate transaminase (AST): 69 IU/L; alanine aminotransferase (ALT): 105 IU/L). An incisional skin biopsy and pathology report revealed subacute vacuolar interface dermatitis with evident basal vacuolopathy, scattered suprabasal keratinocyte apoptosis and mild superficial perivascular mixed lymphohistiocytic and few eosinophilic infiltrates. Moreover, the blood smear revealed atypical lymphocyte (1%). Based on these findings, the patient was diagnosed with drug rash with eosinophilia and systemic symptoms (DRESS). Accordingly, vancomycin and metronidazole were discontinued, and systemic antibiotics were switched to teicoplanin and ceftriaxone. The patient was also prescribed systemic and topical steroids. After 4 days of discontinuation of vancomycin, fever subsided. Laboratory data revealed leukocytosis (white blood cell, 18,200/mm^3^), liver function impairment (AST: 203 IU/L; ALT: 365 IU/L) and atypical lymphocyte (4.1%). Gradually, skin lesions improved after a week, and we slowly tapered systemic steroids. A follow-up MRI of the brain performed after 1 month of initial treatment revealed the reduced size of the abscess lesion and minimal oedema. The patient was discharged with stable condition. After 2 months, out-patient department follow-up reported no headache, dizziness, nausea, or vomiting. Another follow-up MRI performed after 4 months revealed residual granulomatous tissue. The patient reported no neurological signs and could return to normal life. After the brain abscess episode, the patient refused biological agents treatment. The arthritis condition was mild and stable. He is now treated with phototherapy and topical calcipotriol ointment.

## 3 Discussion

Adalimumab therapy reduces the levels of TNF-alpha, but exacerbates the risk of opportunistic infections by two-fold.^[1]^ Studies have reported infections of the upper and lower respiratory tract, skin and soft tissue, bones and joints in patients receiving adalimumab therapy.^[2]^ Besides tuberculosis, other infections, including bacterial, viral and fungal infections, have been documented in prior cases receiving adalimumab treatment.^[3]^ However, CNS infections in patients receiving adalimumab treatment are rare.

Reportedly, brain abscess is a rare and life-threatening parenchymal infection,^[4]^ and *Streptococcus* is the leading causative bacteria. Predisposing factors for brain abscess include the contiguous focus of infection (sinusitis or otitis media), hematogenous seeding (endocarditis, pulmonary, abdominal, or skin infection), penetrating head trauma and neurosurgery. In addition, common pathogens of odontogenic infection comprise *Streptococcus* species (spp.), anaerobes, *Staphylococcus aureus* and *Haemophilus* spp. Of note, polymicrobial infection is common in brain abscess.^[5]^ In our case, *S. sanguinis*, *F. nucleatum*, and *P. micra* were cultured from the specimen.

*S. sanguinis*, a pathogen of the normal oral flora, is commonly related to endocardial infections.^[6]^*F. nucleatum* is a non-sporing, anaerobic, fusiform bacilli commonly found in odontogenic infections.^[7]^*P. micra*, a common commensal of oral cavity, has been reported in bone and joint infections following dental procedures. It is a fastidious Gram-positive cocci previously known as *Peptostreptococcus micros*.^[8]^ All three organisms constitute a part of the oral microbial flora, confirming the odontogenic source of infection in our case.

Despite having a periodontal disease for 5 years, our patient only received partial treatment. The last time he visited a dental clinic was 1.5 years ago; later, he refused to continue the treatment because of the procedure-related pain. Thus, the untreated dental infection might have resulted in the formation of frontal lobe brain abscess. In addition, our patient had neither a history of heart disease, otitis media, sinusitis nor brain trauma. Endocardial sonogram also detected no endocardial lesions. Furthermore, the patient had not received any neurosurgeries or cardiac surgeries, and no other source of bacteraemia in the body was detected in urine, blood, and stool culture.

Currently, no data have shown the time-varying risk of patients with psoriatic arthritis receiving adalimumab treatment. An English literature review revealed that the likelihood of developing an infection is high in the first 6 months of initiating anti-TNF agents but declines after 24–36 months in patients with rheumatic arthritis.^[9]^ Our patient presented with odontogenic brain abscess after the third injection of 40-mg adalimumab received at a 2-week interval. Another case reported *Listeria monocytogenes* brain abscess after 17 days of initiating adalimumab treatment in Crohn disease.^[10]^ Seemingly, infections develop more rapidly in the CNS than other systems; however, it cannot be concluded because only three cases have been documented in the literature to date.^[10–12]^ Our patient developed DRESS syndrome while receiving antibiotics. Furthermore, adalimumab has been reported as one of the factors inducing DRESS syndrome along with vancomycin.^[13]^ In our case, perhaps, DRESS was induced by vancomycin, as the discontinuation of the drug alleviated the symptoms.

There were some limitations in our case. We do not have the document of the patient's previous dental record of the severity of periodontitis. And we did not refer the patient to a dentist before adalimumab treatment. The dental condition before treatment was unknown. We do not have straight forward evidence to prove this brain abscess is induced by adalimumab but we strongly believe that by excluding other possibilities, adalimumab is the culprit to induce opportunistic odontogenic infection.

To the best of our knowledge, this is the first case of a patient with psoriatic arthritis developing odontogenic brain abscess after receiving adalimumab treatment. This case report illustrates the risk of brain abscess in patients treated with adalimumab. Such a rare diagnosis must be kept in mind when patients treated with adalimumab present with sudden-onset convulsions.

## Author contributions

**Conceptualization:** Snehal Pandurang Desale.

**Data curation:** Yu-Pei Lo.

**Investigation:** Snehal Pandurang Desale.

**Methodology:** Po Yuan Wu.

**Software:** Snehal Pandurang Desale.

**Writing – original draft:** Yu-Pei Lo, Po Yuan Wu.

**Writing – review & editing:** Yu-Pei Lo, Po Yuan Wu.

Yu-Pei Lo: 0000-0002-5611-1119.

Yu-Pei Lo orcid: 0000-0002-5611-1119.
